# Occupational segregation, gender essentialism and male primacy as major barriers to equity in HIV/AIDS caregiving: Findings from Lesotho

**DOI:** 10.1186/1475-9276-10-24

**Published:** 2011-06-08

**Authors:** Constance J Newman, Linda Fogarty, Lucia Nthabiseng Makoae, Erik Reavely

**Affiliations:** 1 IntraHealth International, 6340 Quadrangle Drive, Suite 200, Chapel Hill, North Carolina, 27517, USA; 2 Jhpiego, 1615 Thames Street, Baltimore, Maryland 21231-3492, USA; 3 National University of Lesotho, P.O. Roma 180, Maseru, Lesotho; 4 Department of Social Medicine, University of North Carolina, Chapel Hill, USA

## Abstract

**Background:**

Gender segregation of occupations, which typically assigns caring/nurturing jobs to women and technical/managerial jobs to men, has been recognized as a major source of inequality worldwide with implications for the development of robust health workforces. In sub-Saharan Africa, gender inequalities are particularly acute in HIV/AIDS caregiving (90% of which is provided in the home), where women and girls make up the informal (and mostly unpaid) workforce. Men's and boy's entry into HIV/AIDS caregiving in greater numbers would both increase the equity and sustainability of national and community-level HIV/AIDS caregiving and mitigate health workforce shortages, but notions of gender essentialism and male primacy make this far from inevitable.

In 2008 the Capacity Project partnered with the Lesotho Ministry of Health and Social Welfare in a study of the gender dynamics of HIV/AIDS caregiving in three districts of Lesotho to account for men's absence in HIV/AIDS caregiving and investigate ways in which they might be recruited into the community and home-based care (CHBC) workforce.

**Methods:**

The study used qualitative methods, including 25 key informant interviews with village chiefs, nurse clinicians, and hospital administrators and 31 focus group discussions with community health workers, community members, ex-miners, and HIV-positive men and women.

**Results:**

Study participants uniformly perceived a need to increase the number of CHBC providers to deal with the heavy workload from increasing numbers of patients and insufficient new entries. HIV/AIDS caregiving is a gender-segregated job, at the core of which lie stereotypes and beliefs about the appropriate work of men and women. This results in an inequitable, unsustainable burden on women and girls. Strategies are analyzed for their potential effectiveness in increasing equity in caregiving.

**Conclusions:**

HIV/AIDS and human resources stakeholders must address occupational segregation and the underlying gender essentialism and male primacy if there is to be more equitable sharing of the HIV/AIDS caregiving burden and any long-term solution to health worker shortages. Policymakers, activists and programmers must redress the persistent disadvantages faced by the mostly female caregiving workforce and the gendered economic, psychological, and social impacts entailed in HIV/AIDS caregiving. Research on gender desegregation of HIV/AIDS caregiving is needed.

## Background

### HIV/AIDS caregiving in sub-Saharan Africa

It is now recognized that women and girls are the principal caregivers in the vast majority of homes and bear disproportionate responsibility for the psychosocial and physical care of family and community members [1-3]. Women's invisible, unremunerated health care burden derives from multiple inequities that must be tackled at the root to meet the growing need for sustainable community and home-based HIV/AIDS care in sub-Saharan Africa. Gender inequities are particularly acute in HIV/AIDS caregiving (90% of which is provided in the home), where women and girls make up the informal (and mostly unpaid) care workforce. It has been estimated that two thirds of primary caregivers in households in South Africa are female, one quarter of whom are over 60 years of age and 7% of whom are under 18 [4]. A 2008 Joint United Nations Programme on HIV/AIDS (UNAIDS) Expert Group Meeting report presented the following illustrative statistics: In South Africa, 91% of caregivers are female and women carry out eight times more care work (for all illnesses) than men; 68% of volunteers working in three districts in Uganda are female; and two thirds of people living with HIV/AIDS in Thailand are nursed at home by a parent, usually their mother [5,6].

Women's greater responsibility for care results in greater social, physical, and psychological stress and lost opportunities for education, careers, and income [7,8]. Further, community health workers and other HIV/AIDS caregivers have found their jobs are not seen as legitimate *vis à vis *the formal health system, resulting in less access to support and training--another inequity [9-11]. Non- recognition of the material needs of the informal HIV/AIDS workforce, such as water, soap, and gloves, also puts the caregivers' safety at risk. It has also been observed that the women and girls engaged in volunteer, unpaid caregiving also experience longer-term disadvantages, having less access to social protections such as insurance, social security, leave, or pensions because these are typically tied to paid employment status [12]. A Zambian observer has noted that "The burden of HIV and AIDS care has dehumanized women; it has feminized poverty and turned women into workhorses in the name of volunteering and caring for the community" [13].

At the time of this study, Lesotho had the third highest HIV prevalence in the world (an overall seroprevalence of 23.2%, predominantly affecting individuals in their sexually and economically productive years with an urban prevalence of 29% and a rural prevalence of 22%) [14] and the fourth highest rate of tuberculosis incidence, with a growing problem of multi-drug-resistant tuberculosis. These high rates have created an enormous caregiving burden on both the formal and informal health workforces. The 2006 Lesotho Network of People Living with HIV/AIDS (LENEPWHA) Five-Year Strategic Plan indicated that the number of people living with HIV/AIDS was high and steadily growing, with about 55% (146,300) of cases in 2005 being females aged 15-49, of which 75% were aged 15-29. The LENEPWHA strategic plan also noted that these women, who were currently caregivers, would experience mortality, which would exacerbate the caregiving burden [15].

### HIV/AIDS caregiving as occupational segregation

Key contributors to this caregiving burden include global health worker shortages and regional migration, economic crisis and structural adjustment policies that have weakened public health and social security systems and transferred caregiving to households and communities and inadequate and delayed donor and government funding for HIV prevention and treatment [16,17]. But at the heart of women's disproportionate, inequitable HIV/AIDS caregiving burden lies gender inequality and the traditional gender roles and stereotypes that come into play in men's and women's responses to the epidemic. When viewed this way, the unequal sharing of responsibilities between women and men results from and is sustained by the widespread existence of gender status beliefs and stereotypes, the difficulties associated with changing norms around the organization of family caregiving [18], inadequacies in policy, program approaches that have left a caregiving void into which women have stepped (or fallen) as almost exclusive default care providers, and a lack of political will.

Men's and boy's entry into HIV/AIDS caregiving in meaningful numbers would offer an important opportunity both to increase the equity and sustainability of national and community-level HIV/AIDS caregiving responses and mitigate health workforce shortages, especially for societies with high HIV prevalence [19-21]. However, the roles currently played by men and boys as providers of such unremunerated and often unsafe care have been poorly documented and understood, including their willingness to perform this job. While there is positive evidence that some men are entering the HIV/AIDS caregiving job with a range of incentives and social supports [22,23], long-term results from interventions to increase their numbers in HIV/AIDS caregiving are still elusive. Men's significant and sustained engagement in caregiving is by no means inevitable since HIV/AIDS caregiving appears to be a gender-segregated occupation. Making an impact in this vital area health workforce planning requires a better understanding of the dynamics of gender segregation.

Gender segregation is a pervasive and widely documented form of social inequality and labour market rigidity in which women and men are expected to work in culturally defined occupational roles dominated by their gender [24,25]. Typically, women are *vertically segregated *and confined to a narrower range of work in marginal, lower-status, and less well-paid jobs. Women often hold caring and nurturing occupations such as nurses, social workers, and teachers and remain *horizontally segregated *from men who are typically concentrated in technical, diagnostic, managerial, or strength-based jobs: scientists, physicians, managers, police officers, fire fighters, coal miners.

Gender segregation is also one of the most profound and enduring dimensions of labour market inequality, compared with segregation by race or class [26]. Its durability reflects two deeply rooted ideological tenets: The first tenet is *gender essentialism*, which posits that men and women have a basic unchanging "essence" so that, for example, women are perceived as more naturally competent in personal service, nurturance, and social interaction, while men are more competent at mechanical or managerial tasks. The second tenet is *male primacy*, which represents men as naturally dominant and more status-worthy than women [27]. An example of male primacy is illustrated in a study that was recently conducted in Soweto, where male respondents stated they did not participate in HIV/AIDS caregiving activities, even when they felt they should, because of the fear that they would lose respect among their peers if they did so [28]. Gender essentialism and male primacy are aspects of stereotyping that operate in gendered social systems which define men and women as different in socially significant ways [29]. Social inequality is organized around these gender differences, in hierarchies where superior and inferior status is attached to men and women through stereotyping. Gender stereotypes contain status beliefs of "different than" and "better than" which attach to the type of work each gender does. These stereotypes and status beliefs act as barriers to women achieving positions of power and to men assuming positions of lesser social significance. By tying supposed innate traits to tasks, gender essentialism creates "occupational ghettos" which impede the crossover of men into female-identified jobs and vice versa [30].

A key challenge for human resources for health and HIV/AIDS program planners and policy-makers, then, is not only to forge multisectoral, multi-level strategies to deal with the structural, economic and political factors that have contributed to the acute HIV/AIDS caregiving burden carried by women and girls, but to understand and anticipate the entrenched tendency of societies to segregate men and women into different work based on gender ideologies and to assign different values to these differences. Early stage work has focused on ways to increase men's participation in a range of activities that are typically female-identified. There is a growing body of evidence related to male stereotyping that suggests that men's gender-related attitudes and practices can change in relatively short periods of time [31]. A recent review of fifty-seven male involvement programmes published by the World Health Organisation found evidence to suggest that some were effective in transforming harmful gender attitudes and behaviour, while many of the other programmes were regarded as promising [32]. However, none of these studies demonstrated how to significantly increase the equal sharing of domestic or caregiving labour, HIV/AIDS- related or otherwise, especially over the long term. This early stage work has focused on more involved or competent fatherhood, partner assistance during obstetric emergencies, improved communication, shared decision-making among partners, increasing FP and condom use, and reported reductions in sexual partners and severe intimate partner violence. Interventions with men are admittedly limited in size, impact and sustainability and have relied on men's voluntary participation [33]. The extent to which gender equality, gender essentialism and male primacy were key targets of these interventions is not clear.

It has been observed that domesticity as an ideology is socially and culturally constructed and closely linked to patriarchy, subordinate/dominant hierarchical positioning in gender power relations, and an artificial private/public distinction that render the domestic roles of mother, wife, and homemaker as key constructions of women's identity in Africa [34]. It thus seems advisable to target feminine as well as masculine stereotypes for change.

In 2008 the Capacity Project partnered with the Lesotho Ministry of Health and Social Welfare in a study of the gendered division of labour and the gender dynamics of HIV/AIDS caregiving in three districts of Lesotho to account for men's apparent absence in HIV/AIDS caregiving and investigate ways in which they might be attracted and recruited into the community home-based care (CHBC) workforce.

## Methods

Lesotho's Gender and Development Policy had recognized the problem of gender stereotyping and recommended that "The government should advocate for the improvement and expansion of gender-sensitive home-based health care service delivery with particular attention to HIV/AIDS affected and infected persons to alleviate the burden of responsibility on women" [35], observers having noted men's *de facto *absence in these services. The study objectives were to determine the perceived need to bring Basotho men (the ethnic group to which all the study participants belonged) into CHBC and support for HIV/AIDS; determine the feasibility of engaging them as providers of CHBC and support for HIV/AIDS, especially the gender-related and cultural factors to be addressed to increase male involvement based on an analysis of gender relations in Lesotho; and identify factors that facilitate or hinder substantive and sustained male involvement in CHBC and support for HIV/AIDS.

Lesotho is a landlocked country within South Africa with a population of 1.8 million. The country has four distinct ecological zones: the lowlands, foothills, mountains and the Senqu valley. A large percentage of the population, 76.2% resides in rural areas, where poverty is most prevalent. The country depends mainly on subsistence farming, manufacturing and remittances from migrant labor in South African mines. Primary health care (PHC) was adopted as a strategy for health service provision in Lesotho in 1979. Nineteen health service areas were delineated on the basis of the catchment populations. The Government of Lesotho also subsidizes the provision of health services by the Christian Health Association of Lesotho (CHAL) and a limited number of other nongovernmental organizations (NGOs). CHAL provides approximately one-third of health care through a network of eight health service area hospitals and 73 health centers (HCs). CHAL facilities tend to be located in sparsely populated, remote and underserved populations. The most basic level of PHC service provision is at the community/village level where an estimated 4,800 volunteer community health workers (CHWs) are the first line health providers [36].

Data collection took place during February and March 2008. To achieve broad stakeholder participation and representation, data were collected in villages, health clinics, and hospitals across three districts in Lesotho chosen to represent two ecological zones (lowland and highland), to contain both rural and urban sites, and to achieve Ministry of Health and Social Welfare and CHAL representation. Accordingly, the study recruited a stratified sample of voluntary, non-project affiliated participants and used qualitative methods, including 25 key informant interviews with village chiefs, nurse clinicians, and hospital administrators and 31 focus group discussions (FGDs) with community health workers, village men and women, ex-miners, and HIV-positive men and women. Focus group participants were recruited at district Health Centers and through interview participants in order to reach both Community Health Workers (CHWs), people living with HIV/AIDS (PLWHA), and community members not directly affiliated with the health center that could be potential patients or CHWs (e.g., ex-miners, community men and women). Of the total 244 study participants, 70% were women (n = 171) and 30% were men (n = 73). Table 1 shows the study respondents and data collection tools.

**Table 1:** Number of study respondents by data collection tool

Level	Key Informants	Number of Key Informant Interviews N = 25	Focus Group Participants	Number of Focus Group Discussions N = 31
District	District administrators (3), district HIV/AIDS officers (2)	5	HIV-positive men	3
			HIV-positive women	2
			HIV-positive both	1

Health Service Area	District public health nurses (3), medical superintendents (2), TB officer, district medical officer, district health inspector, primary health care director	9		

Health Center	Nurse clinicians	5	Community health workers	9

Village	Chiefs	6	Village men (including ex-miners)	8
			Village women	8

The interview and focus group guides were developed specifically for this study to address the study's research questions, pilot tested in a rural village (Semonkong), and revised according to pilot findings. Interview questions focused on the need for, and feasibility of, involving men in CHBC and what factors facilitate or hinder their participation. Interviews and focus group discussions were audiotaped, transcribed, translated, and coded for analysis. Transcripts were imported into NVivo8 qualitative data analysis software and a codebook was developed according to thematic categories deriving from the four study objectives outlined above, and by gender analysis domains, particularly: the beliefs, attributed knowledge, perceptions, and stereotypes regarding men and women in caregiving; household and domestic division of labour and caregiving practices; and the use of time and space, including whether some spaces were off limits to men as HIV/AIDS caregivers. Code categories were also derived from a close reading of themes in respondents' narrative language and concepts to elicit indigenous categories of meaning and local knowledge about gender, caring, and HIV/AIDS. Coding involved breaking interview transcripts into discrete "text units," interpreting their meaning and assigning a code, and deductively and inductively establishing relationships among codes to form constructs. To test the validity of the codebook and coding strategies, preliminary code reports were reviewed and compared to transcripts by alternate study-team members. Concordance of the coding was assessed and inconsistencies resolved through consensus. The results presented here represent the range, including contradictions and surprises that emerged within themes of participant responses and perceptions about gender and caring labor.

The study design and instruments were approved by the Ministry of Health and Social Welfare Human Subjects Review and all participants provided informed consent.

## Results

### Perceived need for men in CHBC

Study participants uniformly perceived a need to increase the number of CHBC providers to deal with the heavy workload from increasing numbers of patients, and that because of insufficient new entries, the current volunteer workforce was overloaded. Some villages had one health volunteer or none at all, leaving large gaps in care provision. Respondents who were themselves CHBC providers noted that it took a great deal of time to care for the sick, leaving little time to earn money or provide for their own families, and that young volunteers often got "fed up" from being overworked and undersupplied. They expressed the need to increase the number of people involved in caring for sick people, including people affected by HIV/AIDS. Statements in the men's groups reflected a dual perception of women's traditional work and circumstances which demand flexibility and cooperation from men. There was recognition on the part of some men that changing times require a change in caregiving practices, illustrated by this observation from one of the focus groups: "In our tradition, there are things which are the responsibility of women, and caring for sick people is one such thing. It is obvious, though, that because of changing times and new diseases, both men and women should unite against HIV/AIDS" (Miners' FGD).

### Gender beliefs, stereotypes and HIV/AIDS caregiving

#### Gendered division of household labour

The study demonstrated differences between beliefs about the appropriate work of men and women and the real gendered division of labour in households, including tasks that may be associated with CHBC. Some male focus group respondents reported high rates of doing household tasks such as obtaining water and firewood, preparing food, and washing clothes. Fewer men reported providing child care and even fewer reported being engaged in caring for the sick in the family, but overall the data showed that men considered themselves to be already involved in some female-identified domestic and household activities associated with CHBC. The data also showed that women engaged in tasks associated with CHBC at higher rates than men. Further, contrary to the widely-held belief that earning money was "men's work," more women than men reported earning money on a regular basis (i.e., were "breadwinners"). Together, the data suggested that there was some gender integration of domestic and household tasks associated with CHBC, which represents an opportunity to reinforce men's practice and skills in these areas.

#### Gender stereotypes and status beliefs related to caregiving

Shared beliefs about essential male and female traits and gender status reinforced the inequitable division of HIV/AIDS caregiving labour in Lesotho. Both male and female respondents cast women as "natural" family caregivers with inherent traits of nurturance, altruism, sympathy, patience, and self-sacrifice that extended into voluntary community care. According to female focus group respondents, women are "able to bring up a child...able to sweet-talk patients," "good at begging...submissively till he does what he is asked to do" and "naturally are kind and patient" (Village women's FGD). According to male key informants, "Women have courage, sympathy, and patience. They do not choose people, but help anybody who needs care" (Village chief interview). Some respondents also recognized that women are socialized to be caregivers in the context of family life, and that men are not, as illustrated by the following: "Women in nature are nurturing. They have the responsibility of bringing up children, taking care of the home...taking care of the sick. But it doesn't mean that men don't equally have the same skills, it's just that they haven't been practicing them.... It is the same as wanting to know why men do not cook. Traditionally, that has always been a woman's role. It does not mean that there is anything to prevent them from doing it. It's just that they don't perceive it as their own" (District hospital superintendent interview). Whether considered innate or by socialization, caregiving was seen as a source of competence, power, and identity for Basotho women, illustrated by the following focus group statement: "Me, as a mother, woman--am in charge of the sick in my household" (Village women's FGD).

In contrast, men are not understood by nature or socialization to be caregivers, illustrated by the following remarks from community focus groups: "Men are people who are angry quickly and they get fed-up" (Village women's FGD); "The male taking care of the sick? They are there but they are very rare" (District HIV/AIDS officer interview); and "Ever since we grew up, we know that sick people have been cared for by women in the families, they are doing it with all their strength. Because it has been like that, men are not interested; if they do they are very few. Men have to go out and bring money home. Their tasks take them away from home" (District nurse interview).

#### Acceptability of men as caregivers

While much of the data reflected both male and female respondents' perceptions that men are capable of doing various CHBC tasks and that men would accept the caregiver role under certain conditions, there was a widely-held perception that the extent to which men would be accepted as caregivers was circumscribed by tradition and especially by culturally-defined "male" traits that were substantially negative. For example, men face traditional prohibitions against entering the room of a nursing mother, providing care to a daughter-in-law, or entering the place of women's traditional dance--spaces where "women need to be alone" (Village women's FGD). Equally important, however, both male and female informants appeared to mistrust men's motives. Female focus group respondents expressed a high degree of anxiety about their bodies being exposed to men, and women's vulnerability to sexual exploitation in the care setting, since men are perceived as capable of dishonesty, hypocrisy, untrustworthiness, and unscrupulousness when it comes to sex. Illustrations of this from the village women's focus group discussions include: "It is not acceptable, really, because men are not trustworthy people because he can pounce upon you irrespective of your condition, no matter how sick you are;" "Men have promiscuous minds. They think of nothing but adultery when they see women;"and "It is because men misbehave, that is how God has created them, he is capable of making a mistake and mention that he is helping the female patient."

The study found that men who would participate in CHBC might also face contempt and suspicion from both men and women that their motives are really to "womanize." When asked what might be some positive or negative reactions to men's participation in CHBC, the following points emerged: "Men are discouraged by being mistaken as lovers to sick women" (Village women's FGD); "...This gentleman you see with us here today has been ridiculed. 'Is it not the one who runs around with women?' They despise him. That's the problem... They speak ill of him saying he a womanizer."(CHW FGD); and "Whose wife or daughter are you going to play with?" (Village men's FGD).

#### Status and HIV/AIDS caregiving

The study also found that HIV/AIDS caregiving is understood to include undesirable and low-status tasks associated with women's labor, as, for example, in this focus group observation: "When my stomach is running a man will not wash those clothes but women can do that. Men run away" (Village men's FGD). Interestingly, first aid experience related to mining in South Africa was a male-identified skill that was perceived (by men) to be technical and superior to women's family-related caregiving since it was associated with saving lives and featured the "masculine" traits of courage, dignity, bravery, and discretion. First aid was seen to involve the control of bleeding and was distinct from women's "natural" caregiving in the domestic sphere. It was something that men did better than women and that gave men superiority (i.e., primacy) over women, as illustrated by the following: "I think men are much better. Women know nothing about 'safety' so we are above them;" and "The same example of controlling bleeding applies here. Women do not know blood vessels, so that is where we become better" (Miners' FGD). Caregiving *as first aid *was a source of competence and superiority, apparently compatible with the gender identity of Basotho men who had worked in the South African mines.

Apart from its voluntary and low-status nature, study respondents pointed to the role of public ridicule in suppressing men's public displays of HIV/AIDS caregiving. For example, "Some people in the villages ridicule them, saying they are inquisitive and pretending to be nurses *who are not paid*, who cannot provide medication for them" (Public health nurse interview). Peer pressure also stigmatized men who crossed into the "ridiculous" feminine social identity of caregiving and were no longer available for masculine pursuits: "I once heard men ridicule another for taking an 'under five' child for immunizations. 'What have you done with her mother, where is she?' and they were laughing" (PHC director interview). Women's ridicule also suggested that some women might be resistant to men's appropriating women's social identity as caregivers, as when a focus group participant observed that "Other women ridicule them, saying they take women's work and it is theirs" (CHW FGD). It should be noted, however, that other female respondents felt that men's involvement would increase solidarity and ease the caregiving burden.

Nevertheless, the perceived advantages of men as caregivers included men's physical strength to lift, turn, and transport patients, the notions that male patients feel more comfortable being bathed by other men, that men keep secrets, that men communicate better with men, that men have courage and can venture into dangerous places, and that some men are skilled at first aid. There was a strong preference by both men and women for involving men as same-sex providers for men, and for not involving men as providers of intimate care for women outside their families. All this represents an opportunity to bring men into HIV/AIDS caregiving, if they are willing.

### Men's willingness and gendered expectations

Male respondents did not report being unwilling to accept the HIV/AIDS caregiver role but cited as impediments women's traditional caregiving responsibility, their own reticence to intrude on what they perceive as women's preserve of community health work, and their interest in paid work and incentives. Female focus group respondents, including people living with HIV/AIDS and community health workers, also pointed to the fear of stigma as a barrier to men's participation. However, an expectation that men be money-earners served as a prime barrier to men's participation in CHBC, even in times of unemployment. While women may work for free, men will not, and voluntary work such as caregiving is to be avoided because it is inconsistent with an ideal conception of men's status as "breadwinners" (Village men's FGD). This was the case even when women were engaged in breadwinning at higher levels than men, as appeared to be the case in the study sample. Illustrations of the strength of the male breadwinning expectation include the following from the community men's focus group discussions: "The job without incentives is a mockery;" "No man wants to work without incentives;"and "If only some small amount of money could be given, I am sure men would be interested" (Village men's FGD). Other respondents were aware of the importance of pay in men's caregiving: "It looks like men have a problem because caring for the sick is done by volunteers. Men want to be paid for whatever work they do. At present there is no pay. People are working for nothing. That is why they cannot provide care without pay, because they are the ones who fend for the families" (Village women's FGD) and "Men are always breadwinners for their families, so if home-based care is offered free, men will not participate. In fact, the main incentive they are looking for is money. It is very sad when a father gets home at the end of the day claiming to have been doing some work, yet he is not going to get a pay to take care of his family" (Village chief interview). Generally, the study communities appeared to expect women to work for free, in the community as they do at home, and to exempt men from voluntary CHBC work even if they were unemployed and had discretionary time.

### Ways to Increase Men's Participation

The researchers asked focus group participants and key informant interview respondents to suggest ways to increase men's participation in HIV/AIDS caregiving. There appeared to be wide support for the idea that training men could offset the fear that men would sexualize caregiving and increase their acceptability by association with the skill and professionalism of nursing. Examples of this included "You will meet no problem in the village if you help a woman, provided we are trained because you will be going there with your head high as you have the required knowledge for that work" (Village men's FGD); and "What I have observed is that (the) public understands services which include men's involvement yet it used to be women only. In hospitals for instance, people see male nurses, who are even young age-wise. I can only recommend that men be trained well so that they can fit well into the system" (Village women's FGD). Study participants thus appeared to believe that if men could be supervised by a recognized health worker, encouraged, and given financial incentives, they might become more widely accepted as caregivers and more interested in getting involved in HIV/AIDS caregiving. Perhaps most importantly, the Ministry of Health and Social Welfare established a financial incentive for community health workers at $43 per month before the completion of the study, which presumably will attract both more men and women into this job. Table 2 summarizes opportunities and challenges for men's involvement in HIV/AIDS caregiving.

**Table 2:** Opportunities and challenges for men's involvement in HIV/AIDS caregiving

Opportunities	Challenges
Men's physical strength/lifting and transporting patients	Perception of men's dishonesty/womanizing/untrustworthiness/promiscuity
Psychological flexibility	Perception that men will sexualize care situations
Safe mobility	Women's sense of modesty/decorum
Influencing other men to get tested and give care to other men	Traditional prohibitions against entering spaces where women need to be alone; or that father-in-law cannot care for daughter-in-law
Ease of communication with other men	HIV/AIDS caregiving is unpaid labour
Intimate tasks with men	
Sharing and solidarity with women	Male breadwinner status and unwillingness to work without financial incentives
Knowledge of first aid	Men's loss of discretionary time
Men can keep secrets	Appropriation of women's social role
Financial incentive for community health workers	Ridicule

### Results dissemination

Study results were disseminated in a two-day meeting with the Ministry of Health and Social Welfare and other stakeholders in September 2008, including national and district partners, to discuss study findings and validate recommendations for recruiting, training, supporting, and retaining men as HIV/AIDS caregivers in ways that did not disadvantage the women who still carry the burden of care (Some of the recommendations are presented in the "Discussion" section). The meeting included an orientation to gender concepts, presentation of Lesotho's 2003 Gender and Development Policy, and consensus on policy and program actions at national and district levels, elements of which would constitute the foundation of a strategic plan for the national community health worker program. The Ministry of Health and Social Welfare later disseminated results to groups involved in the revision of the national Gender and Development Policy and to the National Curriculum Development Center.

## Discussion

The study results highlighted a nexus of gender stereotypes and status beliefs related to men and women, perceptions of their natural roles, and related social expectations and sanctions that kept women in voluntary HIV/AIDS caregiving and kept men out of it. The findings support the notion that HIV/AIDS caregiving is a gender-segregated job in which unpaid "women's work" in family structures is reproduced as unpaid care for others in the community (e.g., "Ever since we grew up, we know that sick people have been cared for by women in the families, they are doing it with all their strength"). The findings also support the contention that *gender essentialism *and *male primacy *figured in men's striking underrepresentation in HIV/AIDS caregiving activities. Expressions of gender essentialism included "Men are people who are angry quickly and they get fed-up" and "Women in nature are nurturing." There was evidence of what has been referred to as the "discourse of deficient and non-caring men" [37], illustrated by the women's focus group statement that "men are not trustworthy people." At the same time, there was evidence of male primacy, including men's objection to volunteer work ("The job without incentives is a mockery"), the emphasis on the superiority of men's first aid skills ("I think men are much better. Women know nothing about 'safety' so we are above them") and the general expectation that men cannot be expected to work for free (i.e., volunteer work), even when unemployed.

### Can "women's work" be desegregated?

A question must be posed here: What is required to mitigate the gender essentialism and male primacy that sustain occupational segregation in caregiving labour and to develop robust formal and informal workforces capable of providing critical and chronic HIV/AIDS care? Opinion seems to range from pessimism regarding the extent to which women's jobs can be desegregated, to cautious optimism. These perspectives should be kept in mind in HIV/AIDS and health workforce policy, program design, planning and implementation, and research.

"Pessimists" contend that gender stereotypes, status assumptions, and cognitive biases play such a powerful role in the organization of social relations, and that gender is such a fundamental organizing principle of status distribution and inequality in society, that attempts to meaningfully desegregate female-identified health jobs will fail, since men lose respect, status, discretionary time and money for doing "women's work" [38]. The devaluation of women, and by extension, the activities and characteristics associated with women, is deeply inscribed in the cultural norms within a gender hierarchy [39]. There will be "greater resistance to change that involves men taking on traditionally female activities than to change that involves women taking on traditionally male activities" because taking on "women's jobs" typically represents significant losses whereas assuming male activities usually represents an improvement of status for women [40]. A related argument is that both men and women have deep interests in maintaining a clear and reasonably stable framework of gender beliefs and stereotypes that define and differentiate *who *men and women are. In this line of reasoning, gender is so deeply embedded in social relations and institutions that few people are likely to tolerate serious disruptions to the basic system of sex labeling that underpins any gender system [41]. The notion that both men and women have an interest in maintaining a clear framework of gender beliefs and stereotypes appears to be supported by some of the present study results as, for example, in the way public ridicule of men functioned to maintain gender boundaries in caregiving.

However, there is another body of opinion regarding the tractability of gender essentialism, one that might be called cautiously optimistic, which points to the need for strategies to delegitimize the stereotypes that underpin occupational segregation in very specific ways. This body of opinion is represented by what has been referred to earlier as "early stage work" (See the "Background" section). The Lesotho study findings lent themselves to cautious optimism both in female respondents' desire for more solidarity and in the male study respondents' self-reports that they were already assuming female-identified tasks, and that they believed themselves capable of performing or learning CHBC tasks, given training, public legitimacy, supervision and monetary incentives. The study demonstrated that gender segregation was linked to social consciousness (i.e., beliefs), which is amenable to change. Men's psychological flexibility was evidenced by the focus group observation that "In our tradition, there are things which are the responsibility of women, and caring for sick people is one such thing. It is obvious, though, that because of changing times and new diseases, both men and women should unite against HIV/AIDS." The study recommendations therefore included the interventions to act on this flexibility, address resistance to desegregation and delegitimize gender essentialist and male primacy stereotypes. For example:

• Change perceptions of the value (currently low-status) of caring labor and home-based care through a "public relations" campaign that communicates the benefits of HIV/AIDS (and all) caregiving to society or offer public honours and appreciation awards to long-time CHBC providers, with acknowledgment coming from high levels, to create a recognition opportunity that others would strive for.

• Recruit men for caregiving through traditional leaders' courts and means' associations;

• Make HIV/AIDS and domestic caregiving more attractive to men and boys starting with early childhood education and public campaigns that communicate the value of HIV/AIDS (and all) caregiving to society;

• Design training programs that are gender-integrated and that include men's and women's critical thinking about gender roles, stereotypes, and the equal sharing of responsibilities; Introduce male role models already engaged in CHBC. Include mentoring and peer support interventions;

• Review CHBC and primary and secondary school curricula to eliminate gender stereotyping; and

• Offer men and women the same financial and non financial incentives.

Training would presumably render men more socially acceptable as caregivers and bring about changes in men's and women's beliefs and practices. Offering men and financial incentives would presumably render HIV/AIDS caregiving as "breadwinning" for men, while at the same time place value on the "women's work" already done by female caregivers.

### Strategies to increase gender equity in HIV/AIDS programs

There are four roughly discernable strategies that go to the heart of gender inequality and that are useful to consider for increasing gender equity in HIV/AIDS service delivery programs. They all assume the tractability of gender essentialism but differ in the comprehensiveness of approach, focus of interventions and likely effectiveness. Interventions may be included in more than one category. These strategies are summarized in Table 3.

**Table 3:** Strategies to increase gender equity in HIV/AIDS service delivery programs

*Comprehensive*	*Men's participation*	*Women's empowerment*	*Incremental*
Establish a new social consensus that directly addresses the ideology of difference inherent in gender essentialism, and the acceptance of the gender hierarchies inherent in male primacy Target gender power relations and the way in which culture and stereotypes influence unequal sharing of responsibilities On-going public dialogue about and in the sources of gender differences Public policy that stresses gender equality and the sharing of domestic and caregiving responsibility Zero tolerance for the use of stereotypical images of men and women	Promote early, deliberate, and sustained public education and social support regarding the value of caregiving and gender equality Train men and boys to provide care and support Use male role models and recognize men's positive contributions in HIV/AIDS caregiving Focus on engaging men and boys in existing AIDS plans and policies, especially national AIDS plans Improve health systems' capacity to reach men with HIV prevention and treatment services so as to reduce the burden of care Take work with men to scale by integrating a focus on men and gender equality into national programmes and policies that can reach large numbers of men and boys.	Mobilize women's groups and other activists Empower women to effect personal, political and social change Develop critical consciousness to take action against the oppressive elements in one's life, claiming rights, Challenge essentialist notions that unequal gender division of rights and duties is either natural (biological), or God- given or too difficult to change Target gender discrimination patriarchal control of decision-making positions, and patriarchal belief systems Increase access to and control of productive resources Offer comprehensive social protections for women	Involve men in non-personal care tasks or in currently male-identified tasks such as heavy lifting Men and women make small changes in how they as individuals participate in social systems to affect cultural systems in the longer term Recruit men as HIV caregivers

The first strategy is the most comprehensive. It would require establishing a new social consensus that directly challenged the ideology of difference inherent in gender essentialism, and the gender hierarchies inherent in male primacy. This strategy would involve society-wide agreement that socially-constructed gender differences are counterproductive and that it is especially desirable to eliminate essentialist beliefs [42]. This strategy would also require a "far-reaching set of reforms and innovative measures...to address the inequalities that pervade the distribution of responsibilities" [43]. The strategy calls for immediate, concerted and direct efforts to change the social systems that produce these inequalities, since "the problem isn't how we train children to fit into the world; the problem is the world into which we fit them into" [44]. An example might include bringing to scale a mass mobilization technique such as Stepping Stones, which has been widely used in sub-Saharan Africa to transform social norms and relationships around HIV/AIDS, reproductive health, gender-based violence and alcohol abuse. Through gender and age group dialogues, the Stepping Stones approach enables "the possibility of reconciliation between those in society wanting change and those resisting change, so that women and men are engaged together in the process of transformation of gender roles and relations" [45]. While a strategy that aims for changing social consensus might be the most effective in achieving long-term gender equity in the region, arriving at such a consensus would require considerable resources, political and social will and great acumen to counter likely strong resistance.

Less daunting is a second, men's participation strategy, which involves early, deliberate, and sustained public education and social support regarding the value of caregiving and gender equality, use of male role models and the recognition of men's contributions as a way of shifting social norms and prompting other men to do the same, men-inclusive policies and political and civil society activism to change current attitudes and practice in favor of men's and women's equal sharing of the burden of HIV/AIDS care. This strategy draws on recent, early stage work promoting male involvement in parenting, reproductive health and family planning. It is reasonable to suppose that this early stage work may have some impact on men's participation in HIV/AIDS caregiving, but less reasonable to suppose that gender equity will increase in meaningful, sustainable ways if programmes do not directly address the gender essentialist and male primacy ideologies that underpin the gender segregation in HIV/AIDS caregiving. This strategy also seems limited in terms of its power to change gender power relations and the social construction of women's gender identity, an aspect that needs to be considered given the positive social value the Basotho women and men placed on women's caregiving.

The authors call attention to the need to explore women's internalization of gender essentialist and male primacy notions, and their experiences of caregiving inequalities and attempts to share the burden, points often lost in the discussion of men's participation in caregiving. This suggests a third, women's empowerment strategy that is both promising and problematic, given that "the quadruple burden placed by AIDS care on women - weaker health, social exclusion, lack of education and reduced economic power - makes it more difficult for women to advocate for change and engage in efforts to transform their lives and communities. Women's engagement in advocacy and activism is crucial to achieving gender equality, yet those who are fully occupied caring for relatives who are sick with AIDS are less able to participate in such activism" [46]. Women's empowerment, however, is central to social equality, peace and development [47]. Zambian gender activist Sara Longwe has stressed that the "quiet but determined patriarchal opposition to policies of gender equality" (which she sees as political opposition) requires mobilization for women's empowerment to "overcome the many forms of gender discrimination that stand in the way of development" [48]. Interventions in a women's empowerment strategy should address claims that the "unequal gender division of rights and duties is either natural (biological), or God- given or too difficult to change (claimed to be hopelessly and irretrievably embedded in culture)" [49]. In a women's empowerment strategy, change efforts would explicitly target the underlying causes of the unequal sharing of responsibilities (for example, gender status beliefs) and the development of women's critical consciousness, the outcome of which would be women's and girls' greater ability to claim their social and labour rights. For example, increasing women's self assertiveness to "challenge the dominance of male interests" that puts women at risk has become an important priority for HIV/AIDS work in the Zambian organization Thandizani, which has successful expanded its home-based care program, increased its referrals to health facilities, and brought about a greater reported sharing of household tasks and family responsibilities between husbands and wives [50]. Such an empowerment strategy holds promise for long-term change and greater equity, as evidenced by the civil and political rights movements in India, the United States and South Africa.

Recent work in the sociology of gender is suggests an incremental approach which posits that while patriarchal privilege permits men to avoid domestic and caregiving work, it is nevertheless possible for men and women to make small changes in how they as individuals participate in social systems to affect cultural systems in the longer term [51]. Examples of this incremental strategy might be to involve men in nonpersonal care tasks (such as cooking, obtaining water, or gathering firewood) or in currently male-identified tasks (such as heavy lifting) as initial steps toward a more equal involvement of men and women in all caring tasks. However, this type of incremental action is unlikely to impact the gender segregation of HIV/AIDS caregiving, especially in isolation.

Whatever the strategy or strategies chosen, potential resistance to the desegregation of HIV/AIDS caregiving cannot be ignored. Strategies should target both men and women, at some point and should be as comprehensive as possible. They should challenge gender essentialism, gender status beliefs such as male primacy and the relative power and privilege attached to men and women, starting in childhood. Change strategies should be carefully crafted, implemented, and evaluated using behavioural indicators. Further, change efforts should include government policies and programs that distribute the caregiving burden among families/households, markets, not-for profit and non-profit community organisations and the government [52].

Finally, central to any and all effort to increase gender equity, HIV/AIDS and human resources policymakers, activists and programmers must recognize the gendered nature of CHBC, the inequalities and inequities in caregiving labour, and the attendant psychological, physical, and economic impact on women. Policymakers and program managers must immediately address women's and girls' short- and long-term disadvantage by providing the current female caregiving workforce with access to social protections (such as income replacement, medical benefits, credit towards a pension); offering training and supplies for caregiving services; and committing political will and resources to redress the lost opportunities for education, career, and income entailed by HIV/AIDS caregiving (e.g., build education credits or career progression into volunteer jobs).

### Limitations of the Study

Time and resource constraints limited the information that could be collected. Our sample was a convenience sample, and was not selected randomly. This limits the generalizability of findings. However we did find a convergence of opinion across data collection methods (key informant interview and focus group), regions and informant type. We do not have quantitative and comparative information on male and female labour. In asking why men are not involved in home-based care, we did not measure how many caretaker-headed households there were, nor did we directly measure men's absence in HIV/AIDS caregiving. Therefore, we do not know the extent to which is associated with male out-migration, abandonment, death of a spouse or divorce.

#### Further Research

There are several areas of research suggested by this study and by the state of current practice. First, this study brought to the fore cultural prohibitions against men entering a room where a nursing mother resides. Does this prohibition apply to proximity with newborn babies and, if so, what are the implications for men's participation in prevention of mother-to-child transmission services, or in services for the most vulnerable children? Second, though much is presumed, there is little hard evidence on the ways to desegregate male- and female-identified jobs. Research is therefore needed in the area of gender desegregation in order to increase gender equity in HIV/AIDS caregiving. Table 4 summarizes a few possible lines of further program and policy research.

**Table 4:** Lines of further research: Major questions

Do prohibitions against men entering a room where nursing mothers reside apply to proximity with newborn babies and, if so, what are the implications for men's participation in prevention of mother-to-child transmission services, or in services for most vulnerable children?
What is the potential for social and global media to change attitudes and norms around men's caregiving and the equal sharing of domestic labour, especially among children and young men and women who are targeted by early stereotyping?

What are the features of a multisectoral approach that successfully reduces gender essentialism?

What social interventions change gender status beliefs for long-term reductions in gender inequality?

What methods make unpaid, invisible labour and its costs more visible to policymakers? What methods demonstrate the contribution of women's unpaid, invisible caregiving labour to national budgets?

What is better practice in gender equality-oriented caregiving policy?

What is the effectiveness of quotas for male caregivers?

What are the politics of and resistance to equal sharing of responsibilities in a given setting?

What is the effectiveness of school programs that provide role models and caregiving skills to boys?

What strategies and conditions are effective in raising female caregivers' critical consciousness and mobilizing women as agents of change for increased gender equity in HIV/AIDS caregiving?

## Conclusions

HIV/AIDS caregiving is an occupationally segregated job, at the core of which lie notions of gender essentialism, male primacy and gender inequality. There is evidence to support a range of views about the possibility of changing these conditions. If there is to be more equitable sharing of the HIV/AIDS caregiving burden and any long-term solution to health worker shortages, health and HIV/AIDS policy-makers, planners, and human rights activists need to directly address these core gender issues in their strategies.

## Abbreviations

CHAL: Christian Health Association of Lesotho; CHBC: Community and Home-Based Care; CHW: Community Health Worker; FGD: Focus Group Discussion; HC: Health Center; NGO: Non-Governmental Organization; PLWHA: People Living with HIV/AIDS; PHC: Primary Health Care; UNAIDS: United Nations Programme on HIV/AIDS.

## Competing interests

The authors declare that they have no competing interests.

## Authors' contributions

CJN and LF conducted the formative research; conceptualized the study; designed, oversaw, and gave input to all aspects of methodology, data analysis, interpretation, dissemination, and report writing; and reviewed and revised this publication. LNM managed the study in Lesotho. ER analyzed data and reviewed and revised the publication. All authors read and approved the final manuscript.

## References

[B1] Lindsey E Hirshfeld M Tlou S Home-based care in Botswana: experiences of older women and young girls Health Care Women Into 2003 24 6 486 501 10.1080/07399330390199384 12851169

[B2] Ogden J Simel E Grown C Expanding the care continuum for HIV/AIDS: bringing carers into focus Health Policy Plan 2006 21 5 333 342 10.1093/heapol/czl025 16940299

[B3] Sen G Östlin P George A Unequal, Unfair, Ineffective and Inefficient Gender Inequity in Health: Why It Exists and How We Can Change It 2007 Geneva: World Health Organization Commission on Social Determinants of Health

[B4] Steinberg M Johnson S Schierhout G Ndegwa D Hitting Home: How Households Cope with the Impact of the HIV/AIDS Epidemic--a Survey of Households Affected by HIV/AIDS in South Africa 2002 Washington: Henry J. Kaiser Family Foundation

[B5] United Nations Division for the Advancement of Women The Equal Sharing of Responsibilities between Women and Men, Including Caregiving in the Context of HIV/AIDS: Report of the Expert Group Meeting, October 6-9, 2008 2008 New York

[B6] Peacock D Weston M Men and Care in the Context of HIV and AIDS: Structure, Political Will and Greater Male Involvement 2008 Geneva Division for the Advancement of Women Expert Group Meeting on "Equal sharing of responsibilities between women and men, including care-giving in the context of HIV/AIDS."

[B7] Lindsey E Hirshfeld M Tlou S Home-based care in Botswana: experiences of older women and young girls Health Care Women Into 2003 24 486 501 10.1080/07399330390199384 12851169

[B8] Sen G Östlin P George A Unequal, Unfair, Ineffective and Inefficient Gender Inequity in Health: Why It Exists and How We Can Change It 2007 Geneva 10.1080/17441690801900795 19288339

[B9] Ogden J Simel E Grown C Expanding the care continuum for HIV/AIDS: bringing carers into focus Health Policy Plan 2006 21 333 342 10.1093/heapol/czl025 16940299

[B10] George A Human Resources for Health: A Gender Analysis 2007 Geneva

[B11] Reichenbach L ed Exploring the Gender Dimensions of the Global Health Workforce 2007 Boston, Global Equity Initiative, Harvard University

[B12] Sen G Östlin P George A Unequal, Unfair, Ineffective and Inefficient Gender Inequity in Health: Why It Exists and How We Can Change It 2007 Geneva 10.1080/17441690801900795 19288339

[B13] Voluntary Services Overseas (VSO) Reducing the Burden of Care of HIV&AIDS Care on Women and Girls VSO Policy Brief 2006 London 221615934

[B14] Ministry of Health and Social Welfare, Bureau of Statistics, ORC-Macro Demographic and Health Survey (DHS) 2005

[B15] The Lesotho Network of People Living with HIV/AIDS Five Year Strategic Plan for 2006/7-2010/11 2006

[B16] Voluntary Services Overseas (VSO) Reducing the Burden of Care of HIV&AIDS Care on Women and Girls VSO Policy Brief 2006 London21615934

[B17] Peacock D Weston M Men and Care in the Context of HIV and AIDS: Structure, Political Will and Greater Male Involvement 2008 Geneva Paper presented at the Division for the Advancement of Women Expert Group Meeting on "Equal sharing of responsibilities between women and men, including care-giving in the context of HIV/AIDS"

[B18] Daly M The equal sharing of responsibilities between women and men, including care-giving in the context of HIV/AIDS 2008 Geneva Paper presented at the Division for the Advancement of Women Expert Group Meeting on "Equal sharing of responsibilities between women and men, including care-giving in the context of HIV/AIDS"

[B19] Reichenbach L ed Exploring the Gender Dimensions of the Global Health Workforce 2007 Boston, Global Equity Initiative, Harvard University

[B20] Peacock D Weston M Men and Care in the Context of HIV and AIDS: Structure, Political Will and Greater Male Involvement 2008 Geneva Division for the Advancement of Women Expert Group Meeting on "Equal sharing of responsibilities between women and men, including care-giving in the context of HIV/AIDS"

[B21] United Nations Division for the Advancement of Women The Equal Sharing of Responsibilities between Women and Men, Including Caregiving in the Context of HIV/AIDS: Report of the Expert Group Meeting, October 6-9, 2008 2008 New York

[B22] Peacock D Weston M Men and Care in the Context of HIV and AIDS: Structure, Political Will and Greater Male Involvement 2008 Geneva Division for the Advancement of Women Expert Group Meeting on "Equal sharing of responsibilities between women and men, including care-giving in the context of HIV/AIDS"

[B23] Esu-Williams E Schenk K Motsepe J Geibel S Zulu A Involving Young People in the Care and Support of People Living with HIV and AIDS in Zambia 2004 Washington

[B24] Anker R Theories of occupational segregation by sex Int Labour Rev 1997 136 315 339

[B25] Anker R Malkas H Korten A Gender-Based Occupational Segregation in the 1990s 2003 Geneva

[B26] Scott AM ed Gender Segregation and Social Change: Men and Women in Changing Labour Markets 1994 Oxford, Oxford University Press

[B27] Charles M Grusky DB Occupational Ghettos: The Worldwide Segregation of Women and Men 2004 Stanford, Stanford University Press

[B28] Peacock D Weston M Men and Care in the Context of HIV and AIDS: Structure, Political Will and Greater Male Involvement 2008 Geneva Division for the Advancement of Women Expert Group Meeting on "Equal sharing of responsibilities between women and men, including care-giving in the context of HIV/AIDS"

[B29] Ridgeway CL Gender, status and leadership Journal Social Issues 2001 57 637 655 10.1111/0022-4537.00233

[B30] Charles M Grusky DB Occupational Ghettos: The Worldwide Segregation of Women and Men 2004 Stanford, Stanford University Press

[B31] Peacock D Weston M Men and Care in the Context of HIV and AIDS: Structure, Political Will and Greater Male Involvement 2008 Geneva Division for the Advancement of Women Expert Group Meeting on "Equal sharing of responsibilities between women and men, including care-giving in the context of HIV/AIDS"

[B32] World Health Organization Engaging men and boys in changing gender-based inequity in health: Evidence from programme interventions 2007 Geneva

[B33] Peacock D Weston M Men and Care in the Context of HIV and AIDS: Structure, Political Will and Greater Male Involvement 2008 Geneva Division for the Advancement of Women Expert Group Meeting on "Equal sharing of responsibilities between women and men, including care-giving in the context of HIV/AIDS"

[B34] Tamale S Gender Trauma in Africa: Enhancing Women's Links to Resources 2002 Cairo, Council for the Development of Social Science Research in Africa (CODESRIA) and the Arab Research Centre (ARC) Presentation for African Gender Research in the New Millennium: Perspectives, Directions and Challenges

[B35] Government of Lesotho Gender and Development Policy 2003 Maseru 13

[B36] Lesotho Ministry of Health and Social Welfare Human Resource Needs Assessment Survey 2004

[B37] Barker G Engaging Men and Boys in Caregiving: Reflections from Research, Practice and Policy Advocacy in Latin America 2008 Geneva 2 Division for the Advancement of Women Expert Group Meeting on "Equal sharing of responsibilities between women and men, including care-giving in the context of HIV/AIDS"

[B38] Ridgeway CL Blau FD, Brinton MC, Grusky DB Gender as an organizing force in social relations: implications for the future of inequality The Declining Significance of Gender? 2006 New York: Russell Sage Foundation 265 287

[B39] England P Blau FD, Brinton MC, Grusky DB Towards gender equality: progress and bottlenecks The Declining Significance of Gender? 2006 New York, Russell Sage Foundation 245 264 p.245

[B40] England P Blau FD, Brinton MC, Grusky DB. New York, Russell Sage Foundation Towards gender equality: progress and bottlenecks The Declining Significance of Gender? 2006 245 264 p.253

[B41] Ridgeway CL Blau FD, Brinton MC, Grusky DB Gender as an organizing force in social relations: implications for the future of inequality The Declining Significance of Gender? 2006 New York: Russell Sage Foundation 265 287 p.271

[B42] Charles M Grusky DB Occupational Ghettos: The Worldwide Segregation of Women and Men 2004 Stanford, Stanford University Press

[B43] Daly M The equal sharing of responsibilities between women and men, including care-giving in the context of HIV/AIDS 2008 Geneva 25 Division for the Advancement of Women Expert Group Meeting on "Equal sharing of responsibilities between women and men, including care-giving in the context of HIV/AIDS"

[B44] Johnson AG The Gender Knot: Unraveling Our Patriarchal Legacy 1997 Philadelphia, Temple University Press 123

[B45] Hope R Addressing Cross Generational Sex: A Desk review of research and programs 2007 Population Reference Bureau 6

[B46] Peacock D Weston M Men and Care in the Context of HIV and AIDS: Structure, Political Will and Greater Male Involvement 2008 Geneva 4 Division for the Advancement of Women Expert Group Meeting on "Equal sharing of responsibilities between women and men, including care-giving in the context of HIV/AIDS"

[B47] United Nations Fourth World Conference on Women Beijing Declaration and Platform for Action: Action for Equality, Development and Peace 1995

[B48] Longwe SH Addressing Rural Gender Issues: A Framework for Leadership and Mobilisation 2002 Madrid 2 Paper presented at the III World Congress for Rural Women

[B49] Longwe SH Addressing Rural Gender Issues: A Framework for Leadership and Mobilisation 2002 Madrid 5 Paper presented at the III World Congress for Rural Women

[B50] International HIV/AIDS Alliance Working with Men, Responding to Gender, Sexuality and HIV 2003 44

[B51] Johnson AG The Gender Knot: Unraveling Our Patriarchal Legacy 1997 Philadelphia, Temple University Press

[B52] Daly M The equal sharing of responsibilities between women and men, including care-giving in the context of HIV/AIDS 2008 Geneva Division for the Advancement of Women Expert Group Meeting on "Equal sharing of responsibilities between women and men, including care-giving in the context of HIV/AIDS"

